# Elevation gradient effects on grassland species diversity and phylogenetic in the two-river source forest region of the Altai Mountains, Xinjiang, China

**DOI:** 10.3389/fpls.2025.1487582

**Published:** 2025-02-04

**Authors:** Jing Che, Mao Ye, Qingzhi He, Guoyan Zeng, Miaomiao Li, Weilong Chen, Xiaoting Pan, Jiaorong Qian, Yexin Lv

**Affiliations:** ^1^ College of Geographical and Tourism, Xinjiang Normal University, Urumqi, China; ^2^ Xinjiang Laboratory of Lake Environment and Resources in Arid Zone, College of Geographical and Tourism, Xinjiang Normal University, Urumqi, China

**Keywords:** biodiversity, species composition, community phylogenetic diversity, community phylogenetic structure, environmental factors

## Abstract

Altitude, as a key environmental factor, shapes the spatial patterns of species diversity, phylogenetic diversity, and community phylogenetic structure. Studying grassland diversity and phylogenetic structure along altitudinal gradients helps clarify how altitude-driven environmental changes influence community assembly, and reveal vertical patterns in community formation. This study examines grasslands at 1300–2500 m elevation in the Two-River Source Forest Area, Altai Mountains, Xinjiang. Six elevation gradients (200 m intervals) were surveyed with 90 grassland quadrats, documenting community characteristics and environmental data. The study analyzes the patterns of species composition, diversity, and phylogeny across different elevation gradients and explores their relationships with key environmental factors. The results indicate that the grassland species composition is dominated by species from the Poaceae, Rosaceae, and Asteraceae families, with *Poa annua* (annual bluegrass) being the dominant species within Poaceae. The species diversity along the elevation gradient exhibits a bimodal trend, with an initial increase, followed by a decrease, another increase, and finally a decline as the elevation rises. In contrast, phylogenetic diversity shows a unimodal pattern, characterized by an initial increase followed by a decline with increasing elevation. Although the phylogenetic structure did not exhibit a significant trend of transitioning from divergence to clustering along the altitudinal gradient, the overall phylogenetic pattern of grassland communities tended toward clustering. Further analysis reveals significant correlations between species diversity and environmental factors such as temperature, precipitation, forest cover, and soil moisture. However, no environmental factors were found to have a significant correlation with the phylogenetic indices.

## 1 Introduction

Species diversity and phylogeny are essential approaches for studying grassland ecosystems, as they are closely interrelated and jointly influence the structure and function of these ecosystems (Forest et al., 2007). Species diversity is one of the core indicators in community ecology (Abrams, 1995), reflecting habitat differences, structural composition, and stability of plant communities (Tilman and Downing, 1994), and is crucial for understanding the mechanisms behind community formation and maintenance (Yang et al., 2002; Ullah et al., 2024). The study of species diversity generally includes three levels: α-diversity, β-diversity, and γ-diversity, with α-diversity and β-diversity being the most commonly used indices (Whittaker, 1960). Community phylogenetic studies integrate species’ evolutionary history into community ecology by analyzing phylogenetic relationships among species to explore factors influencing community assembly (Webb et al., 2002). This approach examines species diversity distribution patterns from a multidimensional perspective (Wang et al., 2003). Phylogeny reveals the interrelationships among species, which in turn affect ecosystem functions (Srivastava et al., 2012). Compared to species diversity, phylogenetic diversity explores the mechanisms of biodiversity maintenance and species coexistence from a historical evolutionary perspective, addressing gaps in understanding the community assembly process in species diversity research, yielding more accurate and profound results (Schweiger et al., 2008; Petchey and Gaston, 2002). Previous studies have mainly focused on species-level diversity maintenance mechanisms (Gong et al., 2019; Han et al., 2022), with fewer studies combining community ecology and phylogeny (Macheroum et al., 2021). Integrating species diversity and phylogeny offers a deeper understanding of plant community assembly and maintenance.

Currently, the main theories regarding plant community diversity patterns and driving forces include niche theory (Vandermeer, 1972) and neutral theory (Hubbell, 2006). The niche theory posits that ecological differences among species form the basis for their coexistence, with species interactions and abiotic environmental filtering playing decisive roles in shaping community diversity patterns (Chase and Leibold, 2003; Kraft et al., 2015). In contrast, the neutral theory assumes that species have identical ecological functions in survival, reproduction, and competition, emphasizing the role of dispersal limitation in the formation of community diversity patterns (Hubbell, 2011). Elevation gradient, as a key factor influencing species diversity, triggers changes in environmental factors such as temperature, precipitation, and soil, which in turn affect species diversity, phylogenetic structure, and diversity (Gairola et al., 2009). By studying species diversity and phylogenetic structure and diversity across different elevations, we can better understand the impact of environmental factors on these ecological characteristics. Despite the close relationship between species diversity and phylogeny (Chao et al., 2014), there is no consistent pattern in their variation with elevation. Lv et al. (2023) study on the phylogenetic structure of the Bayinbuluke meadow plant community found a divergent structure at low and middle elevations, while clustering occurred at high elevations, a conclusion supported by experiments in the Changbai Mountains of China (Qian et al., 2014). However, Wan Jiamin (Wan et al., 2024) research in northwest Yunnan Province found the opposite: clustering at low and middle elevations and divergence at high elevations, with similar results reported in studies of Tanzanian tropical rainforests (Tallents et al., 2005).

Currently, research on the grassland communities in the Two-River Source Nature Reserve mainly focuses on aboveground biomass, spatial patterns of species diversity, the impact of elevation gradients on community composition and diversity, and the effects of grazing on the economic value of grasslands (Wang et al., 2017; Guo et al., 2022; He et al., 2024). However, studies on grassland communities in the Two-River Source Forest Area from a phylogenetic perspective remain scarce. This study focuses on grassland communities at different elevations within the Two-River Source Nature Reserve in the Altai region of Xinjiang. A total of 30 experimental plots were established across six elevation gradients. The main objectives of this study are: (1) to elucidate the characteristics of species diversity and phylogeny of grassland communities across different elevation gradients and their variation with elevation; (2) to identify the key environmental factors influencing species diversity and phylogeny in grassland communities. We hypothesize that: (1) differences in elevation gradients lead to variations in species diversity and phylogeny, as well as differences in species composition across elevation gradients; (2) species diversity and phylogeny are influenced by multiple environmental factors, and the primary environmental factors affecting species diversity and phylogeny differ.

## 2 Research area and research methodology

### 2.1 Overview of the study area

**Figure 1:**
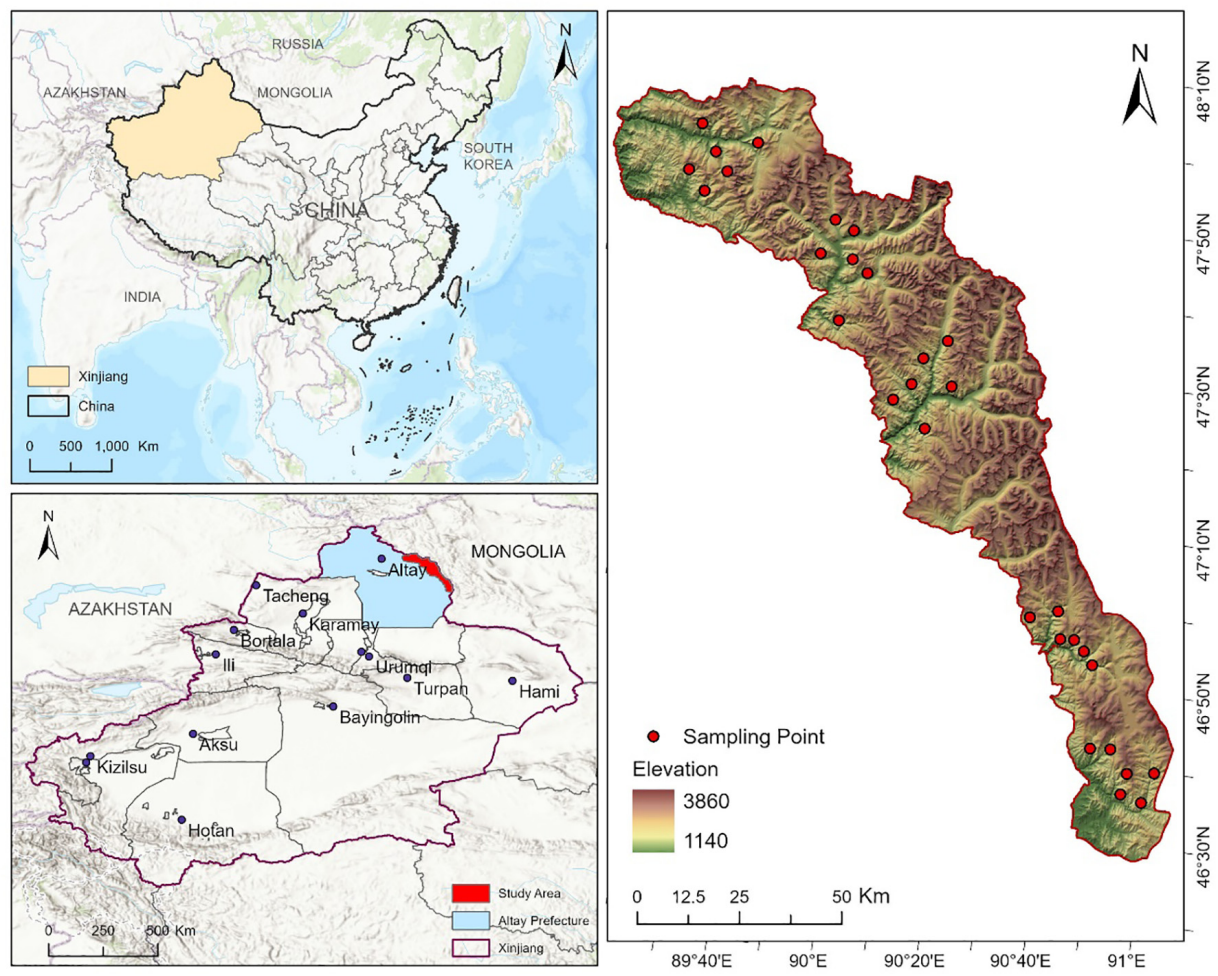
Overview map of the study area.

The Two-Rivers Source Nature Reserve (46° 31’~48° 33’N, 88° 57’~91° 04’E) is located in the northern part of the Xinjiang Uygur Autonomous Region (**Figure 1**), covering a total area of 1.13×10^4 km². The climate of the Two-Rivers Source Nature Reserve is typical of a continental cold temperate climate. The annual average temperature ranges from -2°C to 3°C, with annual precipitation between 100 mm and 300 mm, and an annual evaporation of approximately 1500 mm. The topography is primarily composed of intermountain basins, foreland basins, and river terraces, with well-developed glacial landforms. The terrain is predominantly mountainous, with elevation decreasing from northwest to southeast (Guo et al., 2022). The vegetation in the reserve exhibits significant vertical zonation, with major vegetation types including mountain desert, mountain steppe, mixed coniferous and broadleaf forest, coniferous forest, subalpine meadow, alpine meadow, and alpine tundra (Cao et al., 2020). The reserve’s soil types are diverse, with the main types including brown calcium soil, chestnut calcium soil, mountain gray forest soil, subalpine meadow soil, alpine meadow soil, and mountain marsh soil (Anwar and Abdulla, 2006).

### 2.2 Framework overview

**Figure 2:**
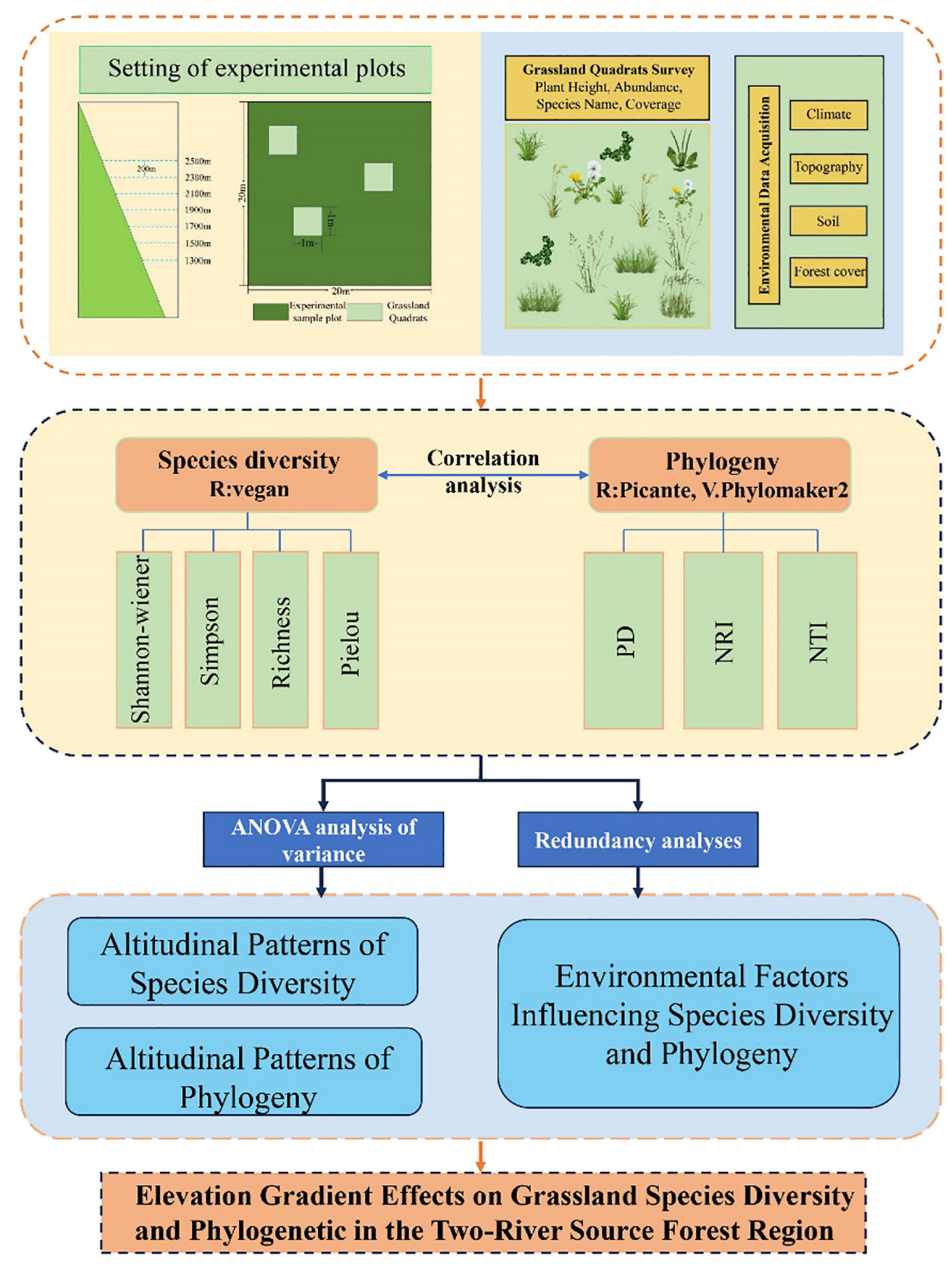
Research framework.

This study systematically analyzed grassland species diversity and phylogenetic structure across different elevation gradients in the Two-River Source Nature Reserve of the Altai Mountains, constructing a comprehensive framework for evaluating grassland ecosystem characteristics and their environmental drivers (**Figure 2**). The study selected six elevation gradients ranging from 1300 to 2500 m (at 200 m intervals) and established a total of 90 grassland quadrats. By integrating species diversity indices (e.g., Shannon and Simpson indices) and phylogenetic metrics (e.g., PD, NRI, NTI), the study examined the patterns of species composition and diversity along elevation gradients and explored their correlations with key environmental factors such as soil moisture, temperature, and precipitation.

In data processing and analysis, species diversity indices were calculated using the vegan package in R, while phylogenetic analyses were conducted using the V. PhyloMaker2 and picante packages to construct a phylogenetic tree and compute relevant indices. Additionally, redundancy analysis (RDA) was employed to evaluate the relationships between diversity indices and environmental factors. Results indicated that species diversity was significantly influenced by environmental factors such as temperature, precipitation, and soil moisture, whereas the patterns of phylogenetic indices showed weaker correlations with these factors. This framework integrates species diversity, phylogenetic analysis, and environmental factor assessment, revealing the multifaceted impacts of elevation gradients on grassland ecosystem characteristics. The study found that species diversity exhibited a bimodal distribution pattern, while phylogenetic diversity followed a unimodal pattern. By synthesizing multiple analytical approaches, this study not only uncovered key mechanisms driving grassland ecosystems but also provided a scientific basis and practical guidance for the conservation and management of grassland ecosystems.

### 2.3 Design of experiments

This study was conducted between June and July 2023, in five regions within the Two-River Source Nature Reserve. Grassland areas within the elevation range of 1300 to 2500 m were selected, with each 200 m increase in elevation delineating an elevation gradient zone, resulting in six elevation gradient zones represented as follows: I (1300-1500 m), II (1500-1700 m), III (1700-1900 m), IV (1900-2100 m), V (2100-2300 m), and VI (2300-2500 m). In each elevation gradient (200 m range), five 20 × 20 m experimental plots were established, aiming to cover the entire 200 m elevation range as comprehensively as possible to maximize sampling representativeness. Within each plot, three 1 × 1 m grassland quadrats were randomly set up. For each quadrat, data on elevation, forest cover, slope, latitude, and longitude were recorded, along with information on the grass species present, plant height, number of individuals, and cover percentage. Soil moisture was measured using the drying method, and meteorological data were obtained from the ERA5 dataset (Muñoz Sabater, 2019).

### 2.4 Data processing

#### 2.4.1 Species diversity index

Based on the data collected from each grassland quadrat, the α-diversity was calculated using the ‘vegan’ package in R-4.3.1. The following indices were calculated for each quadrat: species richness (R), Shannon-Wiener diversity index (*H′*), Simpson diversity index (*D*), and Pielou’s evenness index (*J*), In addition to this, we calculated the importance values of each species within each altitudinal gradient(IV), with the formulas as follows (Ma, 1994a; Ma, 1994b):


(1) R=S

(2) $$H'=-\sum (P_{i}*lnP_{i})$$

(3) $$D=1-(\sum (P_{i}^{2}))$$

(4) $$J=\frac{-\sum P_{i}lnP_{i}}{lnS}$$

(5) $$IVi=[(\frac{H_{i}}{\sum H}+\frac{F_{i}}{\sum F}+\frac{C_{i}}{\sum C})/3]\times 100\%$$

#### 2.4.2 Phylogenetic diversity and phylogenetic structure

**Figure 3:**
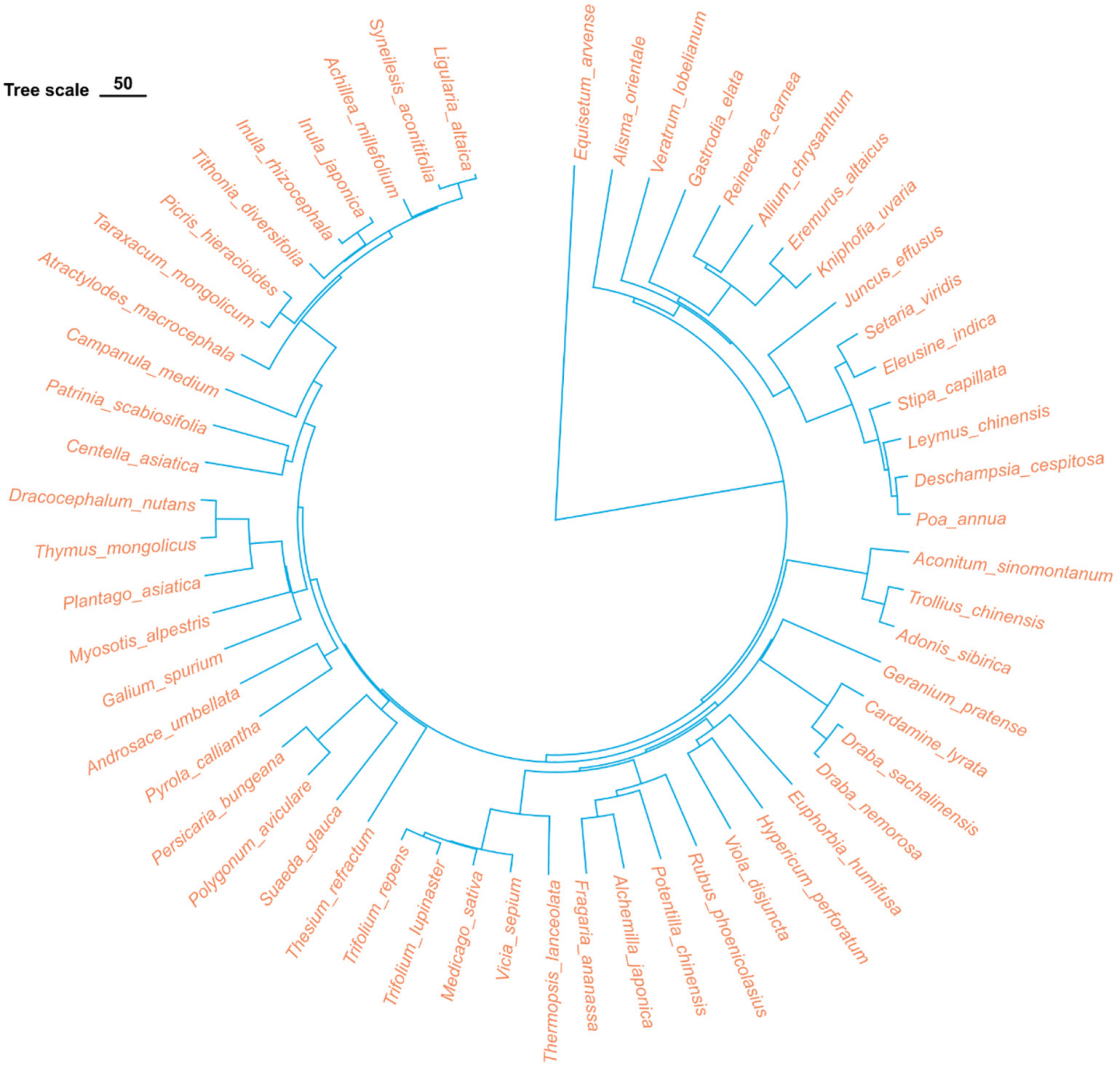
Phylogenetic tree of all plant species in the surveyed samples. The tree was constructed using the V. Phylomaker2 package in R, based on the GBOTB.extended mega-tree.


Based on the species list compiled from the 90 grassland quadrats, the data were organized into a family/genus/species format file, and a phylogenetic tree was constructed using the ‘V. PhyloMaker2’ package in R-4.3.1 (Qian and Qian, 2022) (**Figure 3**). Based on the existing species list, this package employs a predefined phylogenetic framework (GBOTB.extended) to infer phylogenetic relationships based on taxonomic information (family/genus/species). In this study, we used the species list to match taxa with the framework and generate a phylogenetic tree that reflects broad evolutionary relationships among species. Using the synthesized phylogenetic tree file, the phylogenetic diversity index (PD) and phylogenetic structure indices, namely the Net Relatedness Index (NRI) and the Net Nearest Taxon Index (NTI), were calculated using the picante package in R-4.3.1 (Kembel et al., 2010). The calculation formulas are as follows:

(6) $$NRI=-1\times \frac{MPD_{s}-MPD_{mds}}{\text{S}D(MPD_{mds})}$$

(7) $$NTI=-1\times \frac{MNTD_{s}-MNTD_{mds}}{\text{S}D(MNTD_{mds})}$$

Where MPDs represents the observed mean pairwise distance, MPDmds represents the mean of the mean pairwise distances from 999 randomizations, and SD denotes the standard deviation.

### 2.5 Data analysis

The data were initially organized using Excel 2019. One-way analysis of variance (ANOVA) was used to test the significance of differences in species diversity indices and phylogenetic indices across different elevation gradients. The calculation of α-diversity was performed using the vegan package in R-4.3.1 (Dixon, 2003). The construction of the phylogenetic tree was carried out using the V.Phylomaker 2 package in R-4.3.1 (Qian and Qian, 2022), while the calculation of phylogenetic diversity index (PD), and phylogenetic structure indices (NRI, NTI) was conducted using the picante package in R-4.3.1 (Kembel et al., 2010). Pearson correlation analysis was applied to analyze the correlations between species diversity, phylogenetic structure, and phylogenetic diversity. Redundancy analysis (RDA) was conducted using Canoco 5 to explore the relationships between species diversity, phylogenetic structure, phylogenetic diversity indices, and environmental factors.

## 3 Results

### 3.1 Species composition of the community

**Figure 4:**
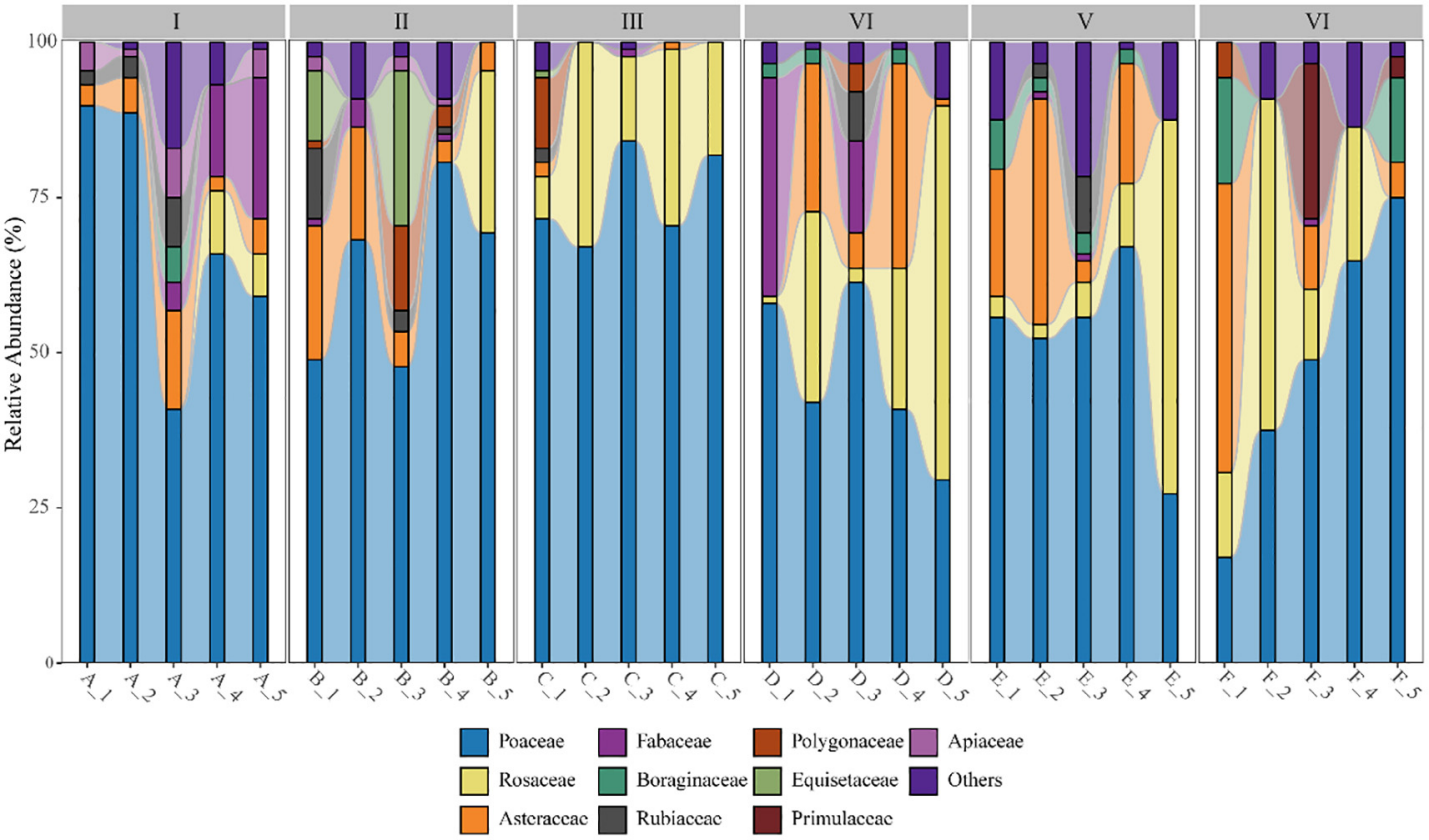
Species composition at different elevations. Elevation gradient I: A_1-A_5; Elevation gradient II: B_1-B_5; Elevation gradient III: C_1-C_5. Elevation gradient IV: D_1-D_5; Elevation gradient V: E_1-E_5; Elevation gradient VI: F_1-F_5.

The collected data from 90 grassland quadrats were sorted, resulting in a total of 57 plant species belonging to 30 families and 54 genera. The relative abundance of various herbaceous plants in the quadrats, categorized at the family level, is shown in **Figure 4**. In terms of species diversity, Elevation Gradient II and Elevation Gradient IV had the highest number of species, with 27 species each, belonging to 16 and 15 families, respectively. Elevation Gradient III had the lowest number of species, with 18 species from 10 families. The trend of species diversity across elevation gradients initially increased, then decreased, followed by another increase, and finally decreased again. Overall, the trend of species diversity along the elevation gradient showed a general decline. Among the 30 grassland samples in the Two-River Source Forest Area, the families Poaceae, Rosaceae, and Asteraceae had the highest relative abundance in the grassland communities.

### 3.2 Importance values of the community

We analyzed the top three species with the highest importance values at each elevation gradient, as shown in **Table 1**. At the species level, the dominant species with higher importance values across the elevation gradients included *Poa annua*, *Deschampsia cespitosa*, *Taraxacum mongolicum*, *Fragaria vesca*, and *Alchemilla japonica*. Among these, *Poa annua*, an annual herb, consistently exhibited the highest importance value across all elevation gradients, with an average value of 0.41 and a peak value of 0.54 at elevation gradient III. Among the top three species with the highest importance values at each elevation gradient, *Poa annua* was the only annual herb, while the other species were perennial herbs. At the family level, the species with the highest importance values across the elevation gradients all belonged to the Poaceae family. Additionally, species from the Rosaceae, Asteraceae, and Fabaceae families also demonstrated relatively high importance values.

**Table 1:** Importance values of species at different altitudinal gradients.

Altitudinal Gradient	Species Name	Family	Life Form	Importance Value
I	*Poa annua*	Poaceae	A	0.34
	*Deschampsia cespitosa*	Poaceae	P	0.37
	*Trifolium repens*	Fabaceae	P	0.17
II	*Poa annua*	Poaceae	A	0.41
	*Deschampsia cespitosa*	Poaceae	P	0.39
	*Taraxacum mongolicum*	Asteraceae	P	0.19
III	*Poa annua*	Poaceae	A	0.54
	*Fragaria vesca*	Rosaceae	P	0.28
	*Stipa capillata*	Poaceae	P	0.19
VI	*Poa annua*	Poaceae	A	0.37
	*Trifolium lupinaster*	Fabaceae	P	0.24
	*Potentilla chinensis*	Rosaceae	P	0.17
V	*Poa annua*	Poaceae	A	0.38
	*Taraxacum mongolicum*	Asteraceae	P	0.16
	*Asphodeline liburnica*	Asphodelaceae	P	0.16
VI	*Poa annua*	Poaceae	A	0.44
	*Alchemilla japonica*	Rosaceae	P	0.26
	*Achillea millefolium*	Asteraceae	P	0.24

A, annual herb; P, perennial herbs.

### 3.3 Species diversity, phylogenetic diversity, and phylogenetic structure as functions of altitude

#### 3.3.1 Species diversity index as a function of altitude

The changes in the three species diversity indices along the elevation gradient in the Two-River Source grasslands are shown in **Figure 4**, and the corresponding species diversity index values are presented in **Table 2**. The Shannon-Wiener diversity index shows that the values are most concentrated in Elevation Gradient III, indicating smaller differences in the Shannon-Wiener index within this gradient. In contrast, the values are most dispersed in Elevation Gradient VI, suggesting larger differences in the Shannon-Wiener index within this gradient. A comparison of the Shannon-Wiener index across the six elevation gradients revealed that the lowest value occurred at elevation gradient III, while the highest value was observed at elevation gradient II. Overall, the Shannon-Wiener index exhibited a bimodal pattern with elevation, initially increasing, then decreasing, followed by another increase and subsequent decrease. The two peaks were found at elevation gradients II and IV. The Simpson index and richness index (R) showed similar differences among elevation gradients and exhibited comparable trends with elevation changes as the Shannon-Wiener index. The Pielou evenness index reached its minimum value at elevation gradient III, but its variation trend with elevation was not distinct.

**Table 2:** Species diversity and phylogenetic structure, phylogenetic diversity at different altitudinal gradients.

Plots	Phylogenetic diversity index	Net relatedness index	Net nearest taxa index	Shannon-wiener index	Simpson index	Pielou index	Richness index
I	611.56 ± 49.86bc	0.16 ± 0.21a	0.78 ± 0.33a	1.13 ± 0.09a	0.54 ± 0.04a	0.6 ± 0.05a	6.6 ± 0.35b
II	985.71 ± 73.13a	-0.57 ± 0.36b	0.08 ± 0.32a	1.14 ± 0.11a	0.53 ± 0.04a	0.63 ± 0.04a	6.8 ± 0.68b
III	679.67 ± 43.3b	0.48 ± 0.11a	0.54 ± 0.18a	0.69 ± 0.08c	0.38 ± 0.04b	0.55 ± 0.05a	3.8 ± 0.34c
IV	577.82 ± 39.26bc	0.24 ± 0.17a	0.76 ± 0.24a	1.11 ± 0.08a	0.57 ± 0.03a	0.66 ± 0.03a	5.53 ± 0.4b
V	669.96 ± 36.35b	-0.09 ± 0.25ab	0.17 ± 0.26a	1.0 ± 0.05ab	0.53 ± 0.03a	0.59 ± 0.03a	6 ± 0.41b
VI	487.65 ± 36.35c	-0.03 ± 0.21ab	0.1 ± 0.21a	0.83 ± 0.13bc	0.43 ± 0.06ab	0.57 ± 0.08a	4.27 ± 0.38c

Different letters in the same column indicated significant differences in species diversity index and phylogenetic index at altitude (P<0.05).

#### 3.3.2 Phylogenetic diversity index and phylogenetic structure index as a function of altitude

**Figure 5:**
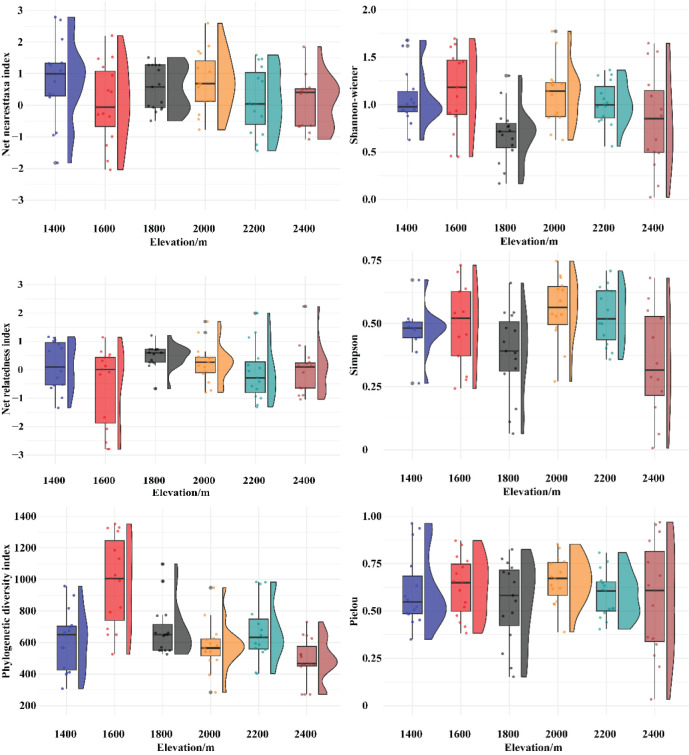
Variation of species diversity and phylogenetic structure index with altitude.

The changes in the phylogenetic diversity index and phylogenetic structure indices along the elevation gradient in the Two-River Source grasslands are shown in **Figure 5**, and the corresponding values are presented in **Table 2**. The phylogenetic diversity index (PD) exhibits a single-peaked pattern, initially increasing and then decreasing with elevation. The net relatedness index (NRI) shows a trend of first decreasing, then increasing, and finally decreasing with elevation, with no clear overall pattern. The highest NRI value occurs in Elevation Gradient III, while the lowest is in Elevation Gradient II. The net nearest taxon index (NTI) shows a general decreasing trend with elevation, with the highest value in Elevation Gradient IV and the lowest in Elevation Gradient II.

### 3.4 Correlation analysis between species diversity, phylogenetic diversity, and phylogenetic structure indices

**Figure 6:**
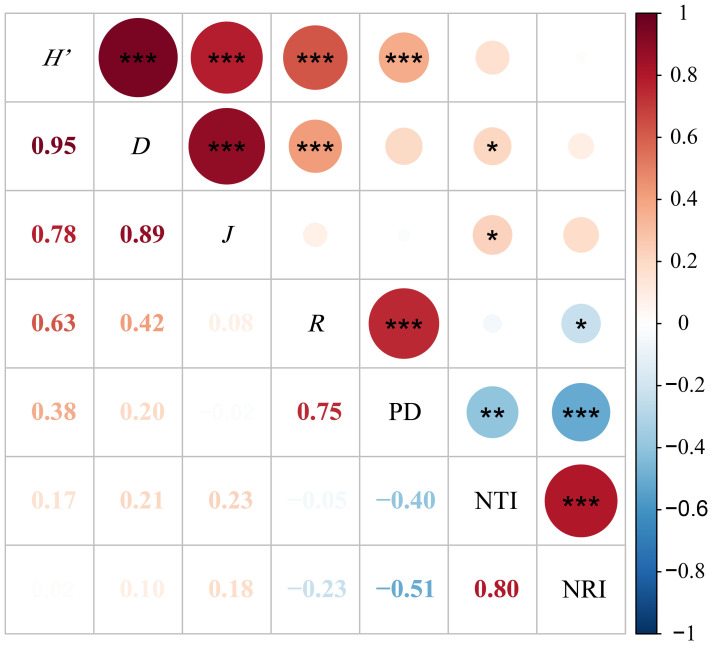
Pearson correlation analysis of grassland species diversity index with phylogenetic diversity index and structural index. *P<0.05; **P<0.01; ***P<0.001. Red indicates a positive correlation, blue indicates a negative correlation, and the darker and larger the circle, the stronger the correlation. PD, Phylogenetic diversity index; NRI, Net relatedness index; NTI, Net nearest taxa index; *H’*, Shannon-Wiener; *D*, Simpson; *R*, Number of species; *J*, Pielou.

Pearson correlation analysis was conducted on the four species diversity indices and the phylogenetic structure indices, as shown in **Figure 6**. The results indicate that the phylogenetic diversity index (PD) is highly significantly negatively correlated with the phylogenetic structure indices NRI and NTI (P < 0.01) and highly significantly positively correlated with the Shannon-Wiener index and SR richness index (P < 0.01). The phylogenetic structure indices NRI and NTI exhibit a highly significant positive correlation with each other (P < 0.01) and a significant negative correlation with the SR richness index (P < 0.05). The NTI is significantly positively correlated with both the Shannon-Wiener index and the Pielou evenness index (P < 0.05). Furthermore, the Shannon-Wiener index shows highly significant positive correlations with the SR richness index, Simpson index, and Pielou evenness index (P < 0.01). Similarly, the Simpson index is highly significantly positively correlated with both the SR richness index and the Pielou evenness index (P < 0.01).

### 3.5 Relationship between environmental factors and species diversity index, phylogenetic structure index and diversity index

**Figure 7:**
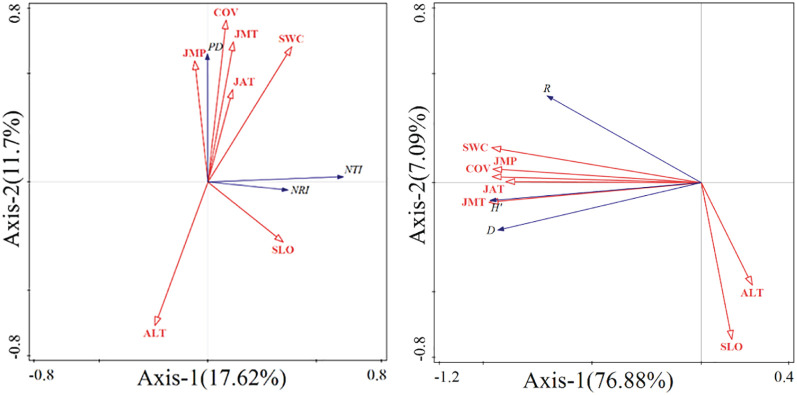
Redundancy analysis of species diversity, phylogenetic diversity, structural diversity and environmental factors.

To explore the reasons for the differences in species diversity, phylogenetic structure, and phylogenetic diversity across different elevation gradients, redundancy analysis was conducted to examine the correlations between species diversity indices, PD index, NRI index, NTI index, and environmental factors. In this study, the indices were used as response variables, and environmental factors were used as explanatory variables. A total of 7 environmental factors, including elevation, forest cover, slope, soil moisture content, and soil temperature, were selected. The analysis results are shown in **Figure 7**.

Regarding the correlation between species diversity and environmental factors, the first and second axes explained a total of 83.96% of the variation. This high percentage indicates that the environmental factors included in the analysis have a strong explanatory power in shaping species diversity patterns. Species diversity indices, including the species richness index (SR), Shannon-Wiener diversity index, and Simpson diversity index, were positively correlated with forest cover, January to May average temperature and precipitation, January average temperature, and soil moisture (p<0.05). In contrast, these indices showed negative correlations with elevation and slope (p<0.05).

The results of the redundancy analysis between the phylogenetic indices and environmental factors show that the first and second axes together explain 29.32% of the variation. However, the analysis results show that the p-values for all environmental factors are greater than 0.05, indicating that they are not statistically significant. Therefore, the environmental factors used in this study do not adequately explain the variation in the phylogenetic indices.

## 4 Discussion

### 4.1 Elevation gradient patterns of species diversity and phylogenetic diversity

Changes in elevation gradients lead to variations in the geographic structure and hydrothermal processes of plant communities and ecosystems, which in turn influence the spatial distribution patterns of species diversity (Ullah et al., 2022). The results of this study indicate that the Shannon-Wiener diversity index and Simpson diversity index exhibit similar trends with elevation, characterized by a bimodal pattern: an initial increase, followed by a decrease, then another increase, and a subsequent decrease, the two peaks were found at elevation gradients II and IV. This result is consistent with the findings of He et al., who studied the effects of elevation gradients on grassland community composition and diversity in the Two-River Source forest region (He et al., 2024). While elevation gradients significantly influence species diversity patterns, human activities further complicate these trends, particularly at mid-elevation gradients. Notably, both the Shannon-Wiener diversity index and Simpson diversity index reached their lowest values at elevation gradient III (1700-1900 m), which deviates from the unimodal patterns reported in previous studies (Lomolino, 2001; Bhattarai and Vetaas, 2003). This discrepancy can be attributed to the higher intensity of human activities at elevation gradient III compared to other elevation sites. Activities such as overgrazing and tourism have caused grassland species loss, leading to a decline in biodiversity (Hou and Yang, 2006; Lu et al., 2011). These activities influence species diversity by altering soil properties, such as increased soil compaction, reduced soil moisture, and decreased vegetation cover, which are commonly associated with overgrazing and trampling (Zhang et al., 2023). Additionally, grazing activities can modify soil microbial communities and alter soil nutrient conditions (Li et al., 2022). As elevation increases beyond gradient IV, human disturbances become minimal, and environmental factors, particularly thermal conditions, emerge as the key determinants of plant growth. Both the Shannon-Wiener diversity index and Simpson diversity index show a decreasing trend at higher elevations, reflecting these constraints (Kraft et al., 2011). The Pielou evenness index also highlights the interplay of elevation and human activities. It generally exhibited a trend of increasing initially and then decreasing with elevation, but a temporary decline was observed at elevation gradient III. This pattern reflects the impact of more intense human activities at this gradient compared to others. Trampling by tourists and livestock, as well as grazing, led to lower species evenness at elevation gradient III. This is likely due to the physical disturbance caused by trampling, which reduces vegetation cover and disrupts the spatial distribution of plant species. Some plant species, particularly those with lower resilience or more specialized habitat requirements, may be more sensitive to these disturbances, leading to a reduction in their abundance and a shift towards dominance by more disturbance-tolerant species. Cheng et al., through an analysis of species diversity indices across different vegetation landscape zones under tourism disturbances in Luyashan, also found a negative correlation between the degree of tourism disturbance and the Pielou evenness index (Cheng and Niu, 2009). These results underscore the dual influence of elevation and anthropogenic pressures on biodiversity patterns.

The phylogenetic community is influenced by multiple factors, including historical evolution, competitive exclusion, and environmental filtering (Wang et al., 2003). In this study, the phylogenetic diversity index exhibited a single-peaked pattern with elevation, with the peak appearing at elevation gradient II. This suggests that the phylogenetic diversity pattern of grassland communities in the Two-River Source region aligns with the mid-elevation bulge pattern, where phylogenetic diversity is highest at mid-elevation regions (Rahbek, 1995). This pattern may be attributed to the combination of increasing precipitation with elevation starting from lower elevations (Anwar and Abdulla, 2006), where favorable hydrothermal conditions promote plant growth and higher species richness. However, as elevation continues to increase, temperature drops, and insufficient thermal conditions lead to lower species diversity in high-elevation plots (Ohdo and Takahashi, 2020), causing the phylogenetic diversity index (PD) to gradually decrease with elevation. This indicates that different environmental factors play key roles in species growth at different elevation gradients. Many previous studies have shown that phylogenetic structure changes with elevation, exhibiting a transition from divergence to clustering (Qian et al., 2014; Xu et al., 2021). In this study, the overall phylogenetic structure was clustered, but no clear trend was observed with elevation changes. We speculate that this phenomenon may be due to the sampling points in this study not covering the entire elevation gradient, resulting in no significant pattern in the phylogenetic structure index with elevation. Notably, in elevation gradient II, the NRI index was divergent, while the NTI index was clustered. The discrepancy between the two indices may be related to the stability of the grassland community (Mastrogianni et al., 2019). This inconsistency may reflect a highly heterogeneous ecological environment, in which different species coexist through various ecological strategies at different spatial and temporal scales (Mastrogianni et al., 2019). There may be competition, suppression, or facilitation relationships that help maintain community stability. Wang et al. found a close relationship between phylogenetic diversity and grassland community stability (Wang et al., 2022). If a community contains species that are sensitive to stress, the loss of these species can lead to a decrease in community diversity and stability, resulting in no apparent clustering or divergence trend in the phylogenetic structure (Li et al., 2021). Therefore, we believe that community structure is not only influenced by environmental factors but also closely related to species interactions and community history (Li et al., 2021). The adaptability of species and niche differences may lead to complex patterns of species distribution in certain environmental conditions.

### 4.2 Correlation of species diversity with phylogeny

Many past studies have demonstrated a significant correlation between community phylogenetic diversity and species diversity (Faith, 1992; Wang et al., 2003). The results of this study also confirm similar conclusions. There is a significant positive correlation between the species richness index and the phylogenetic diversity index(P<0.05), which is consistent with the findings of Li et al. (2021) in their study on species and phylogenetic diversity in Daiyun Mountain. Additionally, research by Lv et al. (2023) in the Bayinbuluk grassland also shows a highly significant positive correlation between species richness and phylogenetic diversity(P<0.01). This is because as species richness increases, a larger number of species leads to increased variability in phylogenetic diversity (Wang et al., 2003), thereby enhancing the phylogenetic diversity of grassland communities. In this study, there is a highly significant negative correlation between phylogenetic diversity and phylogenetic structure(P<0.01), which aligns with findings from other grassland studies in Xinjiang (Shi et al., 2022; Lv et al., 2023). Furthermore, the NTI index shows significant correlations with dominance and evenness indices(P<0.05), indicating that dominance and evenness, in addition to representing species diversity, can also describe the community’s species structure to some extent.

### 4.3 Relationship between environmental factors and species diversity index, PD index, NRI index and NTI index

The results of this study indicate that there is a correlation between species diversity, phylogenetic diversity, and environmental factors. Compared to phylogenetic diversity, the relationship between species diversity and environmental factors is more pronounced. The Simpson index and Shannon-Wiener index show strong positive correlations with forest cover and early growing season temperatures. These results are further supported by the redundancy analysis (RDA), which revealed significant positive correlations between species diversity indices and both forest cover and early growing season temperatures. In areas with higher vegetation coverage, habitat structure is generally more complex, and this environmental complexity determines the diversity of microhabitats, further influencing resource distribution and niche diversity (Currie, 1991). Increased diversity not only affects species composition and coexistence but also shapes the overall biodiversity pattern. Bai et al. pointed out in their study of northern China grasslands that seasonal temperature variations, especially temperatures during the early growing season, have significant impacts on herbaceous plant growth and community diversity (Bai et al., 2022). Suitable temperatures can promote plant germination and growth, thus increasing community species diversity. The RDA results also confirmed that soil moisture and previous year’s temperature significantly contribute to the variation in species richness. Soil moisture directly affects plant survival and growth and enhances competition and resource acquisition capabilities, thereby increasing species richness (Ji et al., 2022). Research by Shi et al. in tropical and subtropical forests also shows that soil moisture has an important impact on species richness by regulating plant growth and resource use efficiency (Shi et al., 2024). At the same time, suitable average temperatures from the previous year may provide favorable conditions for plant germination and survival in the following year, thus increasing species richness (Li et al., 2020). However, with increasing altitude, temperatures gradually decrease and available resources diminish, leading to a significant decline in species richness (Lu et al., 2011; Sharma et al., 2019). Steeper slopes increase environmental heterogeneity, making it difficult for some species to adapt, further reducing species richness. The Shannon-Wiener index is positively correlated with January average temperature and precipitation from January to May. Ye et al.’s research shows that the average precipitation and temperature during key growth periods (including the months before the growing season) have significant effects on grassland ecosystem species richness and diversity, and suitable climatic conditions help maintain higher species diversity (Ye et al., 2024).

The results of the redundancy analysis indicate that the environmental factors selected in this study do not adequately explain the differences in phylogenetic indices. We believe that phylogenetic indices may be more strongly influenced by other environmental factors, such as soil factors. This view has been supported by many previous studies (Wang et al., 2024; Lv et al., 2023). Among them, soil nutrient content is a key factor influencing species diversity, with carbon, nitrogen, and phosphorus being essential elements for plant growth (Yang et al., 2002). Wang et al., in their study on phylogeny at different elevations in the Huo La Mountain of Xinjiang, found a significant positive correlation between soil total nitrogen and the NRI and NTI indices (Wang et al., 2024). This result was also confirmed in the study by Han on the Tibetan Plateau (Han et al., 2022). When nitrogen content is higher, the phylogenetic structure tends to be clustered, which may be due to similar physiological and ecological traits, as well as similar environmental requirements, causing closely related species to occupy similar ecological niches. In addition to nitrogen, phosphorus is also a critical factor influencing community phylogeny. In their study in the northeastern Tibetan Plateau, Xu et al. demonstrated a significant positive correlation between soil total phosphorus and the PD index (Xu et al., 2021), while research by Lv et al. showed a significant negative correlation between soil total phosphorus and the NRI and NTI indices (Lv et al., 2023). Soil total phosphorus affects community phylogeny by altering plant growth conditions, resource acquisition ability, and competition dynamics. Besides the aforementioned soil nutrients, soil moisture is also a key factor affecting community phylogeny (Li et al., 2021). Although this study included soil moisture as an environmental factor, the results did not show a significant correlation between soil moisture and phylogeny. We speculate that this may be because most of the experimental plots in this study are located in the understory grasslands, where soil moisture content differences are small, thus leading to discrepancies between our results and those of previous studies.

### 4.4 Limitations of the current study and future improvement plans

We conducted a study on grassland species diversity and phylogeny across different elevation gradients in the Two-River Source Nature Reserve. However, the current research has certain limitations that warrant further improvement in future studies. Firstly, a broader range of elevation gradients should be selected to enable a more comprehensive and scientifically robust analysis of the effects of elevation gradients on grassland community assembly. Meanwhile, we find that most research indicates that soil factors significantly influence community phylogeny (Wang et al., 2024; Lv et al., 2023). Therefore, in future research, more detailed measurements of soil factors should be conducted to identify the key factors influencing community phylogeny. In addition, conducting studies from the perspectives of different seasons or years represents another direction for future research improvements (Ali et al., 2024). Such analyses could examine grassland dynamics and their underlying drivers across varying temporal scales, thereby providing valuable insights and recommendations for the management of grassland ecosystems.

## 5 Conclusions

This study systematically analyzes the effects of elevation gradients on species composition, species diversity, and phylogenetic diversity in grasslands, and further reveals the key environmental factors driving these changes. The findings contribute to a deeper understanding of species diversity and phylogenetic patterns in the Two-River Source Forest Area. The study found that species diversity changes with elevation in a bimodal pattern, Phylogenetic diversity shows a unimodal pattern with elevation. Phylogenetic structure does not show a significant trend with elevation but generally shows a clustering state in grassland communities. Additionally, species diversity is significantly correlated with environmental factors, with temperature, precipitation, forest cover, and soil moisture content having significant effects on species diversity.

Future research should further consider the effects of soil physicochemical properties on grassland communities and explore the functional diversity of grasslands to obtain more comprehensive and scientific conclusions.

## Data Availability

The raw data supporting the conclusions of this article will be made available by the authors, without undue reservation.
